# Refugee Children’s Social–Emotional Capacities: Links to Mental Health upon Resettlement and Buffering Effects on Pre-Migratory Adversity

**DOI:** 10.3390/ijerph182212180

**Published:** 2021-11-19

**Authors:** Ruth Speidel, Emma Galarneau, Danah Elsayed, Shahdah Mahhouk, Joanne Filippelli, Tyler Colasante, Tina Malti

**Affiliations:** 1 Centre for Child Development, Mental Health, and Policy, Department of Psychology, University of Toronto Mississauga, Mississauga, ON L5L 1C6, Canada; emma.galarneau@mail.utoronto.ca (E.G.); shahdah.mahhouk@mail.utoronto.ca (S.M.); joanne.filippelli@utoronto.ca (J.F.); tyler.colasante@mail.utoronto.ca (T.C.); tina.malti@utoronto.ca (T.M.); 2 Department of Family Relations and Applied Nutrition, University of Guelph, Guelph, ON N1G 2W1, Canada; delsayed@uoguelph.ca

**Keywords:** refugee children, adverse life experiences, social–emotional development, mental health, internalizing symptoms, externalizing symptoms

## Abstract

Refugee children who experience severe pre-migratory adversity often show varying levels of mental health upon resettlement. Thus, it is critical to identify the factors that explain which refugee children experience more vs. less healthy outcomes. The present study assessed child social–emotional capacities (i.e., emotion regulation, sympathy, optimism, and trust) as potential moderators of associations between child, parental, and familial pre-migratory adversities and child mental health (i.e., internalizing and externalizing symptoms) upon resettlement. Participants were *N* = 123 five- to 12-year-old Syrian refugee children and their mothers living in Canada. Children and mothers reported their pre-migratory adverse life experiences, and mothers reported their children’s current social–emotional capacities, internalizing symptoms, and externalizing symptoms. Greater familial (i.e., the sum of children’s and their mother’s) pre-migratory adversity was associated with higher child internalizing and externalizing symptoms upon resettlement. Higher emotion regulation and optimism were associated with lower internalizing and externalizing symptoms, and higher sympathy was associated with lower externalizing symptoms. In contrast, higher trust was associated with higher internalizing symptoms. Finally, higher child optimism buffered against the positive association between familial pre-migratory adversity and child internalizing symptoms. In sum, select social–emotional capacities may serve as potential protective factors that support mental health and buffer against the deleterious effects of pre-migratory adversity in refugee children.

## 1. Introduction

By the end of 2020, over 82 million people had been forcibly displaced across the globe, 42% of whom were children in sensitive stages of development [1]. The refugee crisis is particularly stark in Syria, where political violence, starvation, and other dangers have forced more than half of Syria’s pre-war population to flee since 2011 [1]. Prior to their resettlement, refugee children and families often experience severe adversity (e.g., exposure to war-related violence, family separation) that puts them at risk for elevated emotional and behavioral maladjustment [2]. However, some children exposed to severe adversity show remarkable growth as they adjust to, and even thrive in, difficult contexts [3]. A better understanding of the factors that facilitate mental health upon resettlement and guard against the effects of pre-migratory adversity is critical to inform translational intervention efforts aimed at supporting refugee children’s mental health and positive development. Social–emotional capacities, such as emotion regulation and sympathetic concern for others, have been shown to support child mental health amid risks and adversities outside of the refugee context [4,5], and may extend to protect refugee children from the negative impacts of pre-migratory adverse experiences. The current study assessed several core social–emotional capacities (i.e., emotion regulation, sympathy, optimism, and trust) as moderators of associations between child, parental, and familial pre-migratory adversities and child mental health (i.e., internalizing and externalizing symptoms) upon resettlement in Syrian refugee children living in Canada.

### 1.1. Multiple Levels of Adversity: Child, Parental, and Familial

Developmental psychopathology, ecological systems, and intergenerational trauma theories highlight the importance of considering factors that span the child’s whole experience [2,6,7]. This includes considering functioning across nested familial connections that may contribute to child well-being upon resettlement. For example, in addition to refugee children’s own direct exposure to negative life events, parental exposure to pre-migratory adversities is a risk factor for child mental health (e.g., through parents’ mental health challenges and psychological distress) [8,9,10]. However, in some cases, direct or familial exposure to adversity does not predestine children to mental health challenges, and may even lead to post-traumatic growth and positive development [11]. Examining the impacts of child adversity and parental adversity, respectively, as well as their cumulative effects (i.e., familial adversity), may provide more insight into the differential impacts of early adversity on child mental health in the refugee context. For example, family systems theory suggests that the effects of familial adversity may play a more prominent role in children’s mental health and well-being than direct or parental adversity alone, because this form of risk cuts across multiple ecological levels—the entire family unit’s ability to support child mental health is jeopardized [12]. However, limited studies have assessed the separate and cumulative effects of pre-migratory adversity at different levels of refugee children’s ecological systems. In light of this gap, one of the goals of the current study was to test associations between child, parental, and familial pre-migratory adversities and refugee children’s mental health.

### 1.2. Mental Health in Refugee Children: Internalizing and Externalizing Symptoms

Two prominent markers of mental health are internalizing and externalizing symptoms. Internalizing and externalizing symptoms are the emotional and behavioral difficulties that tend to manifest inwards (e.g., anxiety, depression) and outwards (e.g., aggression, hyperactivity or inattention), respectively [13]. An extensive body of literature links exposure to various forms of early adversity with elevated internalizing and externalizing symptoms among the general population [14].

Given the range of potentially traumatic life events that often characterize the refugee experience at the individual and familial levels (e.g., witnessing violence, family separation), refugee children are considered to be at high risk for internalizing and externalizing symptoms [2]. However, despite these risks, the extant research on this matter is mixed. Some studies demonstrate that children and youth with refugee experiences are at elevated risk for mental health challenges, and that this risk increases as a function of exposure to adverse life events [15,16]. This work includes children originating from different countries and in various stages of refugee status (e.g., pre-displacement, currently experiencing displacement, and post-resettlement), and considers refugee children’s mental health based on the severity of their adverse experiences or relative to nonrefugee comparison groups [17,18,19,20]. Notably, where risk does emerge, the body of evidence seems to indicate greater risk for internalizing vs. externalizing symptoms. For example, one study of refugee youth from Syria, Afghanistan, and Iran resettled in Turkey found that almost half of refugee youth qualified for a psychiatric disorder diagnosis—the majority of which were internalizing disorders [21]. Jensen and colleagues (2015) showed that refugee children’s cumulative adversity was associated with higher internalizing symptoms but not higher externalizing symptoms [22]. Importantly, other studies of refugee children report no differences in mental health or even evidence of lower internalizing and externalizing symptoms as a function of adverse life experiences [23], and similar or better mental health among refugee vs. nonrefugee children [24,25].

Thus, although some evidence paints a picture of risk as a factor of adverse experiences among refugee children, other evidence suggests the presence of protective processes, collectively speaking to the heterogeneity of the refugee experience [2,7,13]. In line with these inconsistent findings, the exact determinants of risk and protective factors among refugee youth are not clear. There have been increasing calls to adopt a child-centered, protection-based focus rather than studying the refugee experience from a deficit-based or comparative model [26]. Such approaches promise to better explain heterogeneity among refugee youth because they focus on identifying and building upon unique strengths across multiple levels of the ecology of the individual and they may better inform attempts to advance youth’s healthy development in spite of risk. Many potential sources of protective factors have been theorized in the refugee context and evidence is emerging for such factors at the individual level (e.g., coping strategies, self-esteem), familial level (e.g., attachment, positive parenting), and community level (e.g., community safety, sense of belonging) [27]. The current study builds upon this work by exploring the potential protective roles of social–emotional capacities. Social–emotional capacities have been shown to promote mental health [28], but they have rarely been explored as protective for children in the refugee context.

### 1.3. Social–Emotional Capacities as Potential Sources of Protection Amidst Adversity

Social–emotional capacities are the broad repertoire of skills that enable children to manage emotions in social settings, connect with and care for others, and understand how they relate to others and the world around them [29]. Under typical circumstances, social–emotional skills are linked to better mental health, including lower internalizing and externalizing symptoms (see [30,31] for meta-analyses). Although less studied, social–emotional capacities are also considered critical protective factors in contexts of risk, including among children exposed to various forms of adversity (for reviews, see [4,5]). Clinicians and practitioners recommend the promotion of social–emotional capacities to support refugee children’s outcomes upon resettlement because these factors have been linked to better mental health and positive development in the general population [32,33]. However, there is limited empirical evidence examining the roles of different social–emotional capacities in mental health in the refugee context. The social–emotional capacities that are the focus of the current study—emotion regulation, sympathy, optimism, and trust—were selected because they reflect distinct yet core components of social–emotional development that span its broad definition. Further, these four capacities may be particularly relevant to the refugee context, as detailed in the paragraphs to follow.

Emotion regulation is the ability to control the occurrence, intensity, and expression of one’s emotions and behaviors in order to achieve goals and behave appropriately in one’s environment [34,35]. Meta-analytic evidence identifies emotion regulation as a robust indicator of mental health, including lower internalizing and externalizing symptoms [36]. Growing evidence suggests that having strong emotion regulation skills may also be protective for refugee youth. For example, the use of more positive emotion regulation strategies, such as cognitive reappraisal, was associated with lower internalizing symptoms, externalizing symptoms, and post-traumatic stress in refugee youth [37,38]. Emotion regulation has also been shown to moderate the effects of adverse life events on mental health among community samples of youth [39], but this moderating effect has yet to be evaluated among refugee youth.

Sympathy is the other-oriented capacity to feel concern for others in distress or experiencing negative situations but is distinct from sharing the distress that the needy other shows (i.e., empathy) [40]. Sympathy is considered an important facet of social–emotional development because it connects children to others, and is associated with lower aggression, more prosocial behaviors, and more positive relationships [41,42]. Although sympathy remains largely under-researched in the refugee context, theory suggests that this capacity may serve as an important protective factor among trauma-exposed youth. For example, sympathy may spur post-traumatic growth by fostering supportive relationships through prosocial orientations and lower aggression [43,44]. Some empirical evidence—albeit not in the refugee youth context—seems to support this theory, showing associations between early adversity and increased sympathy, empathy, and prosocial attitudes in adults [45,46,47]. The current study breaks new ground by extending this past work to examine sympathy in relation to concurrent mental health and as a potential buffer against previous adversities among refugee youth.

Optimism is the propensity to believe that positive outcomes will occur in life and in the future [48]. Greater optimism is associated with better mental health, coping, and more positive relationships [48,49]. Optimism has been implicated as a marker of resilience and post-traumatic growth that promotes children’s potential to thrive in contexts of risk, including among refugee children and adolescents [50,51]. For example, one study on refugee adolescents from various countries resettled in the Netherlands showed associations between higher optimism and higher post-traumatic growth and life satisfaction [51]. Qualitative studies have also identified themes of optimism in association with happiness and coping among refugee children and youth [52,53,54]. Thus, optimism may be a particularly important capacity that supports refugee children’s mental health amidst adversity.

Trust is the capacity to expect others to have benign or positive intentions in social interactions [55]. Trust is generally linked with lower internalizing and externalizing symptoms, and with more positive peer interactions [56,57,58]. From an attachment perspective, violations of trust in early life—such as those that may be experienced by refugee children who are at risk for exposure to severe adversity—can impede the development of trust in others, as well as trust in larger systems or organizations (e.g., government, social services) [59]. Clinicians have indicated a high prevalence of attachment-related issues (~39%) among war-affected refugee children, including particular difficulties with trusting others [60]. Qualitative work among refugee youth has further identified themes of distrust towards others, including social workers, mental health services, and peers [61,62]. Although links between dispositional trust and mental health have not been formally assessed in refugee youth, in one longitudinal study of refugee adults resettled in Australia, higher trust was associated with lower symptoms of depression and post-traumatic stress and greater engagement with other communities [63]. Thus, trust may also be an important social–emotional capacity that contributes to mental health in refugee children and protects against the impacts of pre-migratory adversities.

Some perspectives argue that due to their elevated risk for exposure to severe adversity at the child and familial levels, refugee children are prone to challenges in the realm of social–emotional development. Other perspectives suggest that experiences of early adversity can serve as the impetus for post-traumatic growth, including strengthened social–emotional capacities to cope, manage emotions, look forward to the future, and engage with and show compassion for needy others [43,64]. Despite their theorized role in protecting against adversity [28], social–emotional capacities have yet to be tested as moderators of the link between pre-migratory adversities and mental health upon resettlement in refugee youth. Thus, the current study builds upon existing work to assess the extent to which central dimensions of social–emotional development (i.e., emotion regulation, sympathy, optimism, and trust) contribute to mental health upon resettlement and buffer against the aggravating effects of child, parental, and familial adversities.

### 1.4. The Current Study

This study aimed to address two gaps in the literature: (1) the limited understanding of how different levels of refugee children’s ecology (i.e., child, parental, and familial pre-migratory adversities) affect their mental health; (2) limited evidence for the roles of different strengths-based social–emotional capacities in refugee children’s mental health. To address these gaps, three research questions were assessed: (1) How are child, parental, and familial pre-migratory adversities associated with child internalizing and externalizing symptoms upon resettlement? Given the mixed findings in the refugee literature pointing to both risk and protection amidst adversity, and the lack of research accounting for different ecological levels of adversity, we did not form specific a priori hypotheses for these main effects. However, in the event that negative associations between adversity and mental health did emerge, we adopted a family systems approach. Specifically, we expected stronger negative effects for familial (i.e., child and parental) adversity relative to child and parental adversity alone. (2) Are refugee children’s emotion regulation, sympathy, optimism, and trust associated with their internalizing and externalizing symptoms upon resettlement? In line with past literature spanning normative and diverse at-risk samples, we expected that higher social–emotional capacities would be associated with better mental health, although our hypotheses regarding the relative effects of different social–emotional predictors remained open ended. (3) Are associations between child, parental, and familial pre-migratory adversities and child internalizing and externalizing symptoms moderated by child emotion regulation, sympathy, optimism, and trust? In line with the heterogeneity in past literature on the effects of adversity for refugee youth, we hypothesized that moderation would be present such that social–emotional capacities would buffer any adverse effects of pre-migratory adversity on mental health. However, our hypotheses as to which social–emotional capacities would emerge as moderators remained exploratory.

## 2. Materials and Methods

### 2.1. Study Participants

Participants were 124 five- to 12-year-old children (*M* = 7.99, *SD* = 2.26, 51.6% female) and their mothers. Families were recruited at community events (e.g., foodbank, Saturday Arabic school) and through resettlement agencies in a large city in Canada. At the time of data collection (between March 2017 and September 2018), participating refugee families had arrived in Canada within approximately the past 2 years and had been resettled in Canada for an average of 14.3 months (*SD* = 6.3). On average, families had been displaced for 3.5 years (range 1–10 years) in countries including Turkey, Lebanon, Kuwait, and Jordan before resettling in Canada. Mothers’ highest levels of education were reported as 57% elementary school, 11% high school, and 25% college or university (7% chose not to report). Univariate and multivariate outliers were investigated using ±3 *SD* as a guideline and the Mahalanobis distance measure, respectively. This revealed one outlier who was dropped from subsequent analyses, resulting in a final sample of *N* = 123 refugee children and their mothers for all substantive analyses.

### 2.2. Procedure

The study was conducted according to the guidelines of the Declaration of Helsinki and was approved by the University of Toronto’s Social Sciences, Humanities, and Education Research Ethics Board (protocol number 33501). All mothers provided written or recorded oral consent (in cases where the mother was illiterate), and all children provided recorded oral consent. Data was collected via interviews and questionnaires during 1.5 to 2 h visits that occurred either at the refugee family’s home or mosque, depending on the mother’s preference. During the visit, children and mothers reported individually on their pre-migratory adverse experiences. Additionally, mothers reported their children’s social–emotional capacities and internalizing and externalizing symptoms. Interviews with mothers were conducted in Arabic and interviews with children were conducted in either English (2.9%) or Arabic (97.1%), depending on the child’s preference. There were several procedures in place to support participants if they experienced stress or other negative responses during the study. First, during the consent process, participants were informed that they could end the interview at any time and that they could choose not to answer any questions they preferred not to answer. During the interview, if the caregiver or child showed signs of distress or irritation the interviewer reminded them that they could stop any time they wanted to and that they could skip any questions they did not want to answer. Additionally, a list of multilingual community resources and counselling services was provided to participants. Age-appropriate measures were used for children. Young children and parents with literacy challenges received help as appropriate from trained students who were fluent bilingual speakers. All questionnaires were translated into Arabic and back-translated into English according to standard translation practices [65]. For translation discrepancies, translators discussed options with the research team and came to a mutual agreement. After the interview, mothers were debriefed and given a $10 gift card and each child chose a small gift (i.e., book or toy).

### 2.3. Measures

#### 2.3.1. Child, Parental, and Familial Pre-Migratory Adversities

Children and mothers each answered 5 items taken from the Traumatic Stress Questionnaire [66] and the Social Readjustment Rating Scale [67], which were designed to assess exposure to life stress. Items were selected based on developmental appropriateness and their potential applicability to the refugee experience. Specifically, mothers and children answered yes (1) or no (0) to indicate whether stressful events had happened to them in the past (e.g., “Have any of your close family members died?” and “Have you ever been separated from your family?”). Children’s responses were summed to create a child pre-migratory adversity score (range 0–5), mothers’ responses were summed to create a parental pre-migratory adversity score (range 0–5), and children’s and mothers’ responses were summed to create a familial pre-migratory adversity score (range 0–10).

#### 2.3.2. Child Social–Emotional Capacities

Mothers reported on children’s social–emotional capacities using subscales from the validated Holistic Student Assessment [68]. Specifically, mothers completed 3 items assessing emotion regulation (e.g., “gets easily upset” (reverse coded); α = 0.64), 4 items assessing sympathy (e.g., “feels sad when they see signs of sadness in another child”; α = 0.77), 4 items assessing optimism (e.g., “believes that more good things than bad things will happen to them”; α = 0.76), and 3 items assessing trust (e.g., “thinks most people are fair”; α = 0.80). Items were rated on a 4-point scale from 0 (not at all true) to 3 (almost always true). Composite scores were computed for each subscale, with higher scores reflecting higher social–emotional capacity.

#### 2.3.3. Child Mental Health

Mothers rated their children’s internalizing and externalizing symptoms using an Arabic version of the well-validated Strengths and Difficulties Questionnaire (SDQ) [69]. Following the derivation process recommended by the creators of the SDQ [70], the internalizing symptoms variable was computed as an average of the 5-item emotional problems subscale (e.g., “has many worries or often seems worried”) and the 5-item peer problems subscale (e.g., “rather solitary, prefers to play alone”; α = 0.74), and the externalizing symptoms variable was computed as an average of the 5-item conduct problems subscale (e.g., “often fights with other children or bullies them”) and the 5-item hyperactivity or inattention subscale (e.g., “constantly fidgeting or squirming”; α = 0.60). Items were rated on a 4-point scale from 0 (not at all true) to 3 (almost always true). Higher scores reflected higher internalizing and externalizing problems.

### 2.4. Data Analytic Plan

To answer our research questions, we conducted twelve multiple regression models testing the three independent variables (i.e., child, parental, and familial pre-migratory adversities) separately alongside the four social emotional capacities (i.e., emotion regulation, sympathy, optimism, and trust) as separate moderators in relation to the two mental health outcomes (i.e., internalizing and externalizing symptoms). Running separate models for each independent variable and moderator pair allowed us to avoid multicollinearity, maintain model parsimony, and maximize statistical power. Child internalizing and externalizing symptoms were modeled simultaneously and covaried given their large bivariate association (*r* = 0.57, *p* < 0.001). Child age, child gender, and families’ time living in Canada were included as covariates in all models. After conducting the initial separate models, a model building approach was used whereby any significant main and interaction effects detected in the separate models were tested together in a single merged model to provide a more conservative test of their unique contributions.

The average proportion of missing data across variables was 7.5%. Little’s test of missing completely at random (MCAR) was conducted in SPSS Version 26 using the primary study variables and was nonsignificant, χ^2^ (28) = 39.98, *p* = 0.07, suggesting that the missing data did not violate the assumption of missing completely at random. Thus, all regression models were run in Mplus 8 [71] using full information maximum likelihood estimation to estimate missing data, as this method is robust under conditions of MCAR. The MLR estimator was used to provide maximum likelihood estimations with robust standard errors, accounting for any non-normality in the data.

## 3. Results

### 3.1. Descriptive Statistics

Means, standard deviations, minimum and maximum values, and intercorrelations among the study variables are presented in Table 1. Across the sample, 74.5% of children and 92.7% of mothers reported experiencing at least one adverse pre-migratory experience. Specifically, 25.5% of children and 73.2% of mothers reported experiencing the death of a close family member, 12.4% of children and 45.1% of mothers reported experiencing a major injury or illness, 56.1% of children and 36.6% of mothers reported experiencing family separation, and 55.8% of children and 26.8% of mothers reported witnessing violence. Additionally, 3.1% of children reported experiencing the death of a friend and 65.9% of mothers reported experiencing a major change in financial status. Comorbidity of adverse experiences was relatively high, with 57.1% of children and 74.4% of mothers reporting experiencing two or more adverse life events. Notably, higher child emotion regulation and optimism were associated with lower child internalizing and externalizing symptoms, higher sympathy was associated with lower externalizing symptoms, and higher trust was associated with higher internalizing symptoms. The correlation results among the four social–emotional capacities suggest that these constructs reflect interrelated yet distinct dimensions of social–emotional development in refugee children. Specifically, child sympathy, optimism, and trust were moderately positively associated (*r*s = 0.29–0.37, *p*s < 0.01), yet emotion regulation was associated with lower trust. Results from the 12 initial regression models are summarized below (full results are available in the online Supplementary Materials, Tables S1–S4). Full results of the final merged model are presented in Table 2.

### 3.2. Regression Models Predicting Child Internalizing and Externalizing Symptoms

The main effects of child, parental, and familial adversities on child internalizing and externalizing symptoms were assessed. Child pre-migratory adversity was not associated with child internalizing or externalizing symptoms in any of the four child pre-migratory adversity models. Parental pre-migratory adversity was associated with higher child internalizing symptoms in the model where optimism was modeled as a predictor (β = 0.23, *SE* = 0.11, *p* = 0.04), and was significantly or marginally associated with higher child externalizing symptoms across all four parental pre-migratory adversity models (βs = 0.14–0.20, *SEs* = 0.07–0.11, *ps* = 0.02–0.07). Familial pre-migratory adversity emerged as the most robustly and consistently predictive level of adversity—it was significantly or marginally associated with higher child internalizing symptoms (βs = 0.19–0.36, *SEs* = 0.10–0.14, *ps* = 0.003–0.08) and was significantly associated with higher child externalizing symptoms (βs = 0.21–0.29, *SEs* = 0.09–0.12, *ps* = 0.005–0.03) in all four familial pre-migratory adversity models.

The main effects of child social–emotional capacities on child internalizing and externalizing symptoms were also assessed. Higher child emotion regulation was associated with lower child internalizing (βs = −0.50–−0.52, *SEs* = 0.06–0.07, *ps* < 0.001) and externalizing symptoms (βs = −0.56–−0.58, *SEs* = 0.07, *ps* < 0.001) in the child, parental, and familial pre-migratory adversity models. Higher child sympathy was significantly and marginally associated with lower child externalizing symptoms in the parental pre-migratory adversity model (β = −0.20, *SE* = 0.10, *p* = 0.04) and the familial pre-migratory adversity model (β = −0.16, *SE* = 0.09, *p* = 0.08), respectively. Higher child optimism was associated with lower child internalizing (βs = −0.28–−0.33, *SEs* = 0.09–0.10, *ps* ≤ 0.001–0.003) and externalizing symptoms (βs = −0.23–−0.25, *SEs* = 0.09–0.10, *ps* = 0.004–0.01) in the child, parental, and familial pre-migratory adversity models. Higher child trust was associated with higher child internalizing symptoms in the child, parental, and familial pre-migratory adversity models (βs = 0.19–0.29, *SEs* = 0.09, *ps* = 0.002–0.03).

The moderation effects of child social–emotional capacities on associations between child, parental, and familial adversities and child internalizing and externalizing symptoms were assessed. No significant interaction effects emerged in the child or parental pre-migratory adversity models. In the familial pre-migratory adversity models, two significant interactions emerged. First, child emotion regulation moderated the association between familial adversity and child externalizing symptoms (β = −0.14, *SE* = 0.07, *p* = 0.047), in that the association between familial pre-migratory adversity and child externalizing symptoms upon resettlement was significant and positive at lower levels of emotion regulation (β = 0.42, *SE* = 0.14, *p* = 0.004) but was nonsignificant at higher levels of emotion regulation (β = 0.02, *SE* = 0.03, *p* = 0.55). Second, child optimism moderated the association between familial adversity and child internalizing symptoms (β = −0.31, *SE* = 0.13, *p* = 0.02), in that the association between familial pre-migratory adversity and child internalizing symptoms upon resettlement was significant and positive at lower levels of optimism (β = 0.75, *SE* = 0.25, *p* = 0.002) but was nonsignificant at higher levels of optimism (β = −0.03, *SE* = 0.14, *p* = 0.86).

The covariate effects were as follows. Throughout the models, child gender was significantly associated with child externalizing symptoms such that boys were rated higher in externalizing symptoms than girls (βs = 0.18–0.30, *SEs* = 0.07–0.09, *ps* ≤ 0.001–0.05).

Next, we brought the significant main and interaction effects from the previous models together into a single merged model to provide a more conservative test of their robustness, stability, and unique contributions to mental health. Specifically, the previously obtained main effects of familial pre-migratory adversity, emotion regulation, optimism, and trust on internalizing symptoms were examined. Additionally, the main effects of familial pre-migratory adversity, emotion regulation, optimism, and sympathy on externalizing symptoms were modeled. Finally, the interaction between familial pre-migratory adversity and emotion regulation on child externalizing symptoms and the interaction between familial pre-migratory adversity and optimism on child internalizing symptoms were incorporated for evaluation. As in previous models, child age, child gender, and length of stay in Canada were included as covariates. (Albeit significant in the preliminary models, the main effect of parental pre-migratory adversity was dropped from the final merged model because it was highly collinear with familial pre-migratory adversity (*r* = 0.82). Familial pre-migratory adversity was retained because it was the most consistent adversity predictor in the preliminary models. Notably, including both adversity variables did not alter the statistical significance of the other effects in the merged model, with the exception that it diluted the main effect of familial pre-migratory adversity. This is to be expected because of the overlap in their variance explained.) The merged model fit the data well (χ^2^(4) = 6.23, *p* = 0.18, RMSEA = 0.07, CFI = 0.99, SRMR = 0.02). In this model (see Table 2), the interaction between familial pre-migratory adversity and optimism remained statistically significant while controlling for the other significant main effects on internalizing symptoms, although the interaction between child emotion regulation and familial pre-migratory adversity dropped to nonsignificant, suggesting that externalizing symptoms were better informed by the other significant main effects in the model (i.e., child emotion regulation and sympathy).

**Figure 1:**
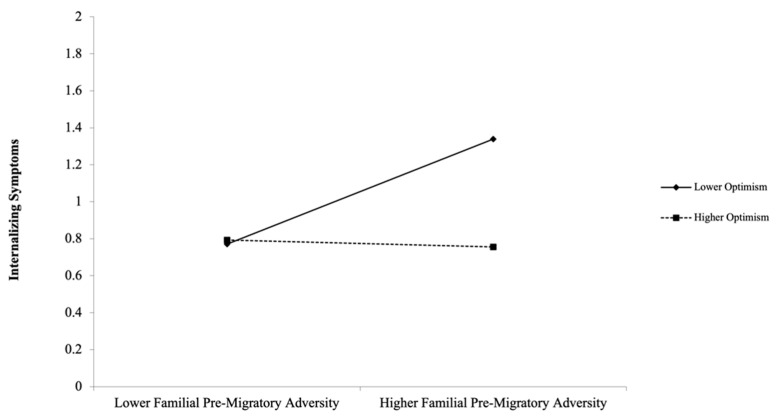
Interaction plot. Familial pre-migratory adversity in relation to internalizing symptoms at lower (−SD) and higher (+1 SD) levels of optimism. The observed range of internalizing symptoms was 0.0 to 1.9.

The effect of familial pre-migratory adversity on child internalizing symptoms was again probed at 1 standard deviation below and above the mean of child optimism. In line with our buffering hypothesis and as depicted in Figure 1, the association between familial pre-migratory adversity and child internalizing symptoms upon resettlement was significant and positive at lower levels of optimism (β = 0.62, *SE* = 0.23, *p* = 0.008) but was nonsignificant at higher levels of optimism (β = −0.04, *SE* = 0.13, *p* = 0.76). Overall, predictors in the final merged model explained 43% of the variance in child internalizing symptoms and 50% of the variance in child externalizing symptoms.

## 4. Discussion

The current study tested the effects of pre-migratory adversities on refugee children’s mental health and considered the potential direct and moderating roles of key social–emotional capacities at the child level. The results add to the literature in three key ways: (1) by identifying familial adversity (as opposed to child and parental adversity alone) as a potential source of risk for children’s internalizing and externalizing symptoms upon resettlement; (2) by revealing associations between distinct social–emotional capacities and mental health in refugee children; (3) by highlighting optimism as a particularly important social–emotional capacity that may protect refugee children from the detrimental effects of pre-migratory familial adversity.

### 4.1. Main Effects of Child, Parental, and Familial Pre-Migratory Adversities on Refugee Children’s Mental Health

Given the potential differential roles of adversity at different levels of refugee children’s ecology [2], the first objective of the current study was to examine the potential effects of child, parental, and familial (i.e., the sum of child and parental) pre-migratory adversities on refugee children’s mental health upon resettlement. Of the three indicators of adversity, familial pre-migratory adversity was most consistently associated with higher child internalizing and externalizing symptoms. Parental adversity was associated with higher child externalizing symptoms but was only associated with higher child internalizing symptoms in one of the four parental adversity models, suggesting that children of mothers who experience more pre-migratory adversities are at higher risk for externalizing vs. internalizing symptoms. Notably, child pre-migratory adversity was not associated with children’s internalizing or externalizing symptoms.

Our results are in line with theories of developmental psychopathology, ecological systems, and intergenerational trauma [2,6,7], which emphasize the importance of considering different contexts of the family experience. They are also in line with literature suggesting that cumulative risks across different ecologies may deleteriously impact child mental health [12]. Cumulative familial adversity played a more salient role in children’s mental health than direct or parental adversity alone. This is in line with past work suggesting that adversity spanning the family system can be most threatening to child mental health, especially in the absence of protective factors [72,73].

The minimal evidence for associations between parental adversity and child internalizing symptoms, as well as the null associations between child adversity and both indices of child mental health, run contrary to some of the past evidence in the literature [73] but align with other past evidence of both risk and protection amidst adversity [11]. Notably, adverse life experiences do not always translate directly into mental health challenges; in fact, some evidence indicates that adversity in the refugee context can result in post-traumatic growth, whereby experiences of adversity lead to better mental health [24]. For children in this study, it is also possible that adversity at one level (e.g., at the child level alone) was countered or offset by a lack of adversity at another level (e.g., at the parental level), which may free up parental resources to support the adversely affected child. This could be the case because even within the same family, individuals may be exposed to unique experiences and adversities that impact their capacity to engage with others. For example, when a parent (or another family member) has experienced severe adversity, the potential stress and impacts on mental health related to processing that trauma may impede their efforts to support a child who has also experienced adversity.

### 4.2. Main Effects of Child Social–Emotional Capacities on Child Internalizing and Externalizing Symptoms

Our second objective was to assess child emotion regulation, sympathy, optimism, and trust in relation to child internalizing and externalizing symptoms. We found that emotion regulation and optimism were each uniquely associated with lower internalizing and externalizing symptoms. Further, sympathy was associated with lower externalizing symptoms. These associations align with theories of post-traumatic growth because they identify associations whereby strengthened social–emotional capacities to cope, manage emotions, look forward to the future, and show concern for others can contribute to mental health in refugee children [43,64]. These results also speak to the importance of considering strengths-based associations in refugee youth [17,32], and align with past studies involving typically developing lower-risk samples or samples exposed to different risk contexts, such as children from low-income homes [74]. Importantly, our results extend the findings of these studies to the refugee context, which adds to existing literature by further suggesting that emotion regulation and sympathy may serve as common themes in mental health (particularly, externalizing symptoms) across populations [75]. The capacity to flexibly regulate emotions may support refugee children’s coping with the challenges and resulting stress of their unique experiences, thereby promoting their internalizing and externalizing symptoms upon resettlement. Further, having the capacity to reflect on the world and personal situations with greater optimism may help refugee children adjust to their new surroundings more quickly and seamlessly. Finally, the current study highlights the potential supportive role of sympathy in the refugee context, suggesting that other-oriented concern may mitigate the risk for externalizing behaviors, such as acting out and harming others, as refugee children resettle. Importantly, emotion regulation, optimism, and sympathy are all moldable capacities [76,77]. The current results suggest that these capacities may be useful ports of entry for intervention efforts aimed at supporting refugee children’s mental health and well-being upon resettlement.

Interestingly, trust was associated with higher internalizing symptoms in the current study, which runs contrary to our hypothesis and past work examining trust in relation to mental health in nonrefugee samples of children [56,57,58]. A consideration of how trust was operationalized in the current study may inform this finding. Specifically, the Holistic Student Assessment uses 3 items to assess trust, namely “thinks most people are fair”, “trusts other people”, and “believes most people can be trusted”. Notably, this metric of trust extends beyond children’s direct acquaintances or close contacts to refer to people in general. This conceptualization could inform the association detected between higher child trust and higher child internalizing symptoms in the current sample. For example, more focused trust within a tighter relational circle (i.e., immediate family, friends, direct community) might be more adaptive in contexts of migration-based adversity, whereas interpersonal trust that extends beyond one’s ingroup may result in elevated risk for those exposed to external violations of trust that are common in such contexts. Relatedly, it is important to consider how trust may function differently in contexts of severe adversity. In the pre-migratory context, as indicated here, refugee children are often exposed to severe traumas, including extreme violence, family separation, and other human rights violations. These violations, especially early in life, can impede children’s development of trust in others [61,62]. In addition, lower trust may function to protect against potential exploitation in unpredictable and difficult environments [78], which may explain why higher trust in the present sample of refugee children was seemingly maladaptive. In other words, we may have tapped into the residual negative effects of being too trusting in a relatively unpredictable and dangerous pre-migratory environment. Possibly, refugee children’s trust may not work in a similarly protective manner to other social–emotional capacities, at least early in the resettlement process. Notably, our bivariate correlation results revealed that higher trust was associated with lower emotion regulation, further suggesting that the capacity for interpersonal trust may work differently among children who have experienced severe interpersonal adversities. While the current results support the specificity principle (i.e., the notion that outcomes will vary depending on the specificities of the group under study) [75], they also align with select past work indicating the limitations of “too much trust” or “too little trust” in typically developing samples. Specifically, some studies have documented a curvilinear association between trust and mental health in children and adolescents, with higher and lower levels of trust being associated with poorer mental health (indexed by both internalizing symptoms and aggression) relative to moderate levels of trust [79,80]. Future work should continue to assess the nature of trust in refugee youth and how this capacity relates to their mental health throughout the resettlement process.

### 4.3. Moderating Effects of Child Social–Emotional Capacities

Of the four social–emotional capacities under study, optimism emerged as the only robust moderator of the link between pre-migratory adversity and child mental health upon resettlement. Specifically, at lower levels of child optimism, higher familial pre-migratory adversity was associated with higher child internalizing symptoms. However, this effect was buffered in that when children were rated as higher in optimism, there was no longer an association between familial pre-migratory adversity and child internalizing symptoms. These results support protective approaches to refugee mental health by identifying optimism as a factor that may protect against the potential harmful effects of pre-migratory adversity on refugee youth. Optimism refers to the propensity to believe that positive outcomes will occur in life and in the future [48]. Greater optimism is associated with better mental health, coping, and more positive relationships [48,49]. Given their risk for exposure to early negative life events that run contrary to an optimistic outlook, refugee children’s optimism may be at risk. However, optimism has been implicated as an important marker of protection and post-traumatic growth that can directly promote children’s potential to thrive in adverse contexts by orienting them away from the lingering effects of past negative events and towards positive future growth [50,54]. Thus, optimism may be particularly helpful for refugee youth to the extent that they can maintain a positive outlook in spite of their challenges. The current study aligns with past qualitative studies of refugee children and youth that identify themes of optimism in association with happiness and coping [52,53,54], and importantly extends this past work by quantitatively identifying optimism as a factor that may protect against the potentially detrimental developmental impacts of familial pre-migratory adversity.

Although optimism has traditionally been considered a stable personality trait, growing work suggests that it can be nurtured and taught, including in refugee youth [81,82]. In one recent study, a brief, strengths-based intervention implemented with children in refugee camps in Greece was successful in increasing children’s optimism, at least in the short term [81]. Notably, cognitive–behavioral therapy (CBT) often employs clinical strategies that are aimed at supporting individuals’ capacities to approach and evaluate situations in a more optimistic manner [77], potentiating the viability of CBT for refugee populations. Overall, our findings suggest that optimism may be a fruitful source of protection for refugee intervention efforts to continue to target.

### 4.4. Limitations and Future Directions

The current study has notable strengths, including its unique sample of Middle Eastern and Syrian refugee families recently resettled in Canada, its use of multiple reporters, its assessment of early adversity across multiple ecological contexts, and its strengths-based focus. Despite these strengths, there are several limitations that bear mention. First, the current study employed a cross-sectional design. Although pre-migratory adversities occurred prior to study enrollment, the current investigation cannot tease apart whether higher social–emotional capacities precipitated children’s experience of better mental health, or vice versa. Although children and mothers each reported their own experiences of adversity, mothers reported on both child social–emotional capacities and mental health. Caregiver reports often provide informative and reliable insights into children’s well-being [83], but less is known about how refugee mothers (who have often experienced severe adversity themselves) evaluate their children’s social–emotional capacities and mental health. Further, there are limitations to relying on mothers’ reports as our metric of the broader family system. Given that the gender of children was associated with some of the study outcomes (i.e., males were rated higher in externalizing symptoms relative to females), it is important to consider that mothers may report differently on their children’s social–emotional capacities relative to other or additional caregivers. Further, the use of a single informant for multiple measures may have introduced some bias to the results, despite our efforts to account for collinearity in our statistical model building. Future work should consider integrating multiple informants as well as observational measures of outcomes.

As is common in the refugee context, a broad range of adverse experiences characterized the current sample. Given large comorbidities between such experiences, we were limited in our ability to evaluate the unique effects of individual adverse experiences. Future work should assess whether there are differential impacts of distinct adversities (e.g., witnessing violence vs. experiencing family separation).

Finally, it is important to note that the present study examined the adversities, social–emotional capacities, and mental health of Middle Eastern and Syrian refugee children resettling in Canada. In line with the specificity principle, the findings should be interpreted with the unique characteristics of our sample in mind. For example, according to Canadian immigration policy at the time of our sample’s immigration, only intact families (including both parents and their dependent children under 19), lone women, and children who entered with a relative or guardian were allowed to seek refuge in Canada [84,85]. In addition, many families in our sample were part of a cohort of refugees that migrated together in unusually high numbers. This may have led to aspects of their everyday resettlement experiences being different from those of other refugees resettling in Canada. For example, their ESL/ELL classes were filled mostly with other Arabic-speaking Syrian students and there were more services initiated and staff hired to cater specifically to the large wave of incoming refugees that they comprised [85,86]. Therefore, the current findings may or may not be generalizable to other refugee contexts. The complementarity principle (i.e., the idea that development is simultaneously characterized by commonalities and specificities) may also be applicable here [75], because the current sample of refugee children may share both common and unique experiences and characteristics with other refugee populations that both bear implications for mental health.

## 5. Conclusions

The current study highlights the importance of key social–emotional capacities for refugee children’s mental health in the context of multiple levels of adversity across the family system. Notably, in the current study higher child emotion regulation, optimism, and sympathy predicted better child mental health upon resettlement. In contrast, higher trust corresponded with higher internalizing symptoms during resettlement, which may speak to the unique pitfalls of high trust within the experience of severe migration-related trauma. Further, higher child optimism buffered against the aggravating effects of familial pre-migratory adversity on child internalizing symptoms. The findings open up several areas of questioning for future research to consider. For example, in line with the specificity principle, are there particular social–emotional capacities that play a role in supporting adjustment amid different forms of refugee-related adversity (e.g., family separation vs. witnessing violence) or different combinations of adverse experiences (e.g., family separation and witnessing violence)? Additionally, do similar associations emerge in different populations of refugee children or among nonrefugee children exposed to adversity (e.g., maltreatment, community violence)? Finally, the current results suggest that it may be advantageous for intervention efforts to evaluate whether supporting core social–emotional capacities (i.e., emotion regulation, optimism) bolsters adjustment among refugee children. In sum, the results of the current study highlight the potential utility of promoting social–emotional capacities to support mental health and positive development in some refugee children.

## Figures and Tables

**Table 1:** Intercorrelations among study variables.

	1.	2.	3.	4.	5.	6.	7.	8.	9.	10.	11.	12.
1. Child pre-migratory adversity	—											
2. Parental pre-migratory adversity	−0.01	—										
3. Familial pre-migratory adversity	**0.57** ***	**0.82** ***	—									
4. Emotion regulation	0.01	−0.05	−0.03	—								
5. Sympathy	−0.20	0.08	−0.09	−0.17	—							
6. Optimism	−0.06	0.15	0.09	0.12	**0.37** ***	—						
7. Trust	**−0.21** *	**0.24** *	0.12	**−0.30** **	**0.29** **	**0.29** **	—					
8. Internalizing	0.02	0.14	0.16	**−0.53** ***	0.11	**−0.28** **	**0.23** *	—				
9. Externalizing	0.19	0.12	0.22	**−0.55** ***	**−0.23** *	**−0.27** **	0.02	**0.57** ***	—			
10. Child age	0.05	0.09	0.09	−0.16	0.14	−0.05	0.07	0.18	−0.04	—		
11. Child gender (male)	0.16	**−0.23** *	−0.08	−0.03	**−0.21** *	**−0.22** *	−0.16	0.10	**0.24** **	0.02	—	
12. Length of stay in Canada	−0.14	**−0.27** *	**−0.29** **	−0.12	0.15	−0.06	0.02	0.04	−0.02	**−0.23** *	0.14	—
Min	0.00	0.00	0.00	0.00	0.75	1.25	0.00	0.00	0.20	5.00	0.00	3.00
Max	4.00	5.00	9.00	3.00	3.00	3.00	3.00	1.90	2.40	12.00	1.00	30.00
*M*	1.41	2.48	3.84	0.97	2.41	2.60	1.94	0.94	1.09	8.01	—	14.34
*SD*	0.99	1.44	1.76	0.72	0.58	0.46	0.75	0.46	0.52	2.26	—	6.30

Note. Statistically significant correlations are denoted in bold; * *p* < 0.05, ** *p* < 0.01, *** *p* < 0.001.

**Table 2:** Merged regression model predicting child internalizing and externalizing symptoms.

	Internalizing			Externalizing		
Variable	β	*SE*	*p*	β	*SE*	*p*
Child age	0.10	0.08	0.19	−0.09	0.07	0.20
Child gender (male)	0.09	0.07	0.22	**0.18**	**0.07**	**0.01**
Length of stay in Canada	0.07	0.07	0.34	−0.01	0.08	0.88
Familial pre-migratory adversity	**0.29**	**0.11**	**0.006**	**0.20**	**0.08**	**0.009**
Emotion regulation	**−0.39**	**0.07**	**<0.001**	**−0.60**	**0.07**	**<0.001**
Optimism	**−0.31**	**0.08**	**<0.001**	−0.07	0.07	0.34
Trust	**0.19**	**0.07**	**0.007**	-	-	-
Familial Pre-Migratory Adversity × Optimism	**−0.26**	**0.12**	**0.03**	-	-	-
Sympathy	-	-	-	**−0.27**	**0.08**	**0.001**
Familial Pre-Migratory Adversity × Emotion Regulation	-	-	-	−0.10	0.07	0.14

Note. Standardized results are presented. Significant effects bolded.

## Data Availability

The data are not publicly available because we do not have ethics approval or consent from the participating families to share this data.

## Supplementary Materials

The following are available online at https://www.mdpi.com/article/10.3390/ijerph182212180/s1, Table S1: Emotion regulation regression models predicting child mental health for each adversity type. Table S2: Sympathy regression models predicting child mental health for each adversity type. Table S3: Optimism regression models predicting child mental health for each adversity type. Table S4: Trust regression models predicting child mental health for each adversity type.
