# Supplementary material for: Refugee Children’s Social–Emotional Capacities: Links to Mental Health upon Resettlement and Buffering Effects on Pre-Migratory Adversity
Source: Int J Environ Res Public Health. 2021 Nov 19;18(22):12180. doi: 10.3390/ijerph182212180 (PMC8618894; doi:10.3390/ijerph182212180)
Supplement: Supplementary file 1 [file ijerph-18-12180-s001.zip › ijerph-1432057-supplementary.pdf]

Table S1. Emotion regulation regression models predicting child mental health for each adversity type

	Internalizing			Externalizing		
	β	*SE*	*p*	β	*SE*	*p*
**Child pre-migratory adversity model**						
Child age	.09	.08	.28	**−.17**	**.07**	**.01**
Child gender (male)	.08	.08	.30	**.22**	**.07**	**.002**
Length of stay in Canada	−.002	.08	.98	−.13	.08	.10
Child pre-migratory adversity	.01	.10	.92	.13	.08	.12
Emotion regulation	**−.52**	**.07**	**<.001**	**−.58**	**.07**	**<.001**
Child Pre-Migratory Adversity x Emotion Regulation	-.02	.08	.85	.01	.08	.85
**Parental pre-migratory adversity model**						
Child age	.09	.09	.30	−.14	.07	.07
Child gender (male)	.11	.08	.17	**.30**	**.07**	**<.001**
Length of stay in Canada	.03	.09	.76	−.11	.08	.14
Parental pre-migratory adversity	.15	.11	.18	**.14**	**.07**	**.05**
Emotion regulation	**−.50**	**.07**	**<.001**	**−.56**	**.07**	**<.001**
Parental Pre-Migratory Adversity x Emotion Regulation	.003	.09	.98	−.13	.07	.06
**Familial pre-migratory adversity model**						
Child age	.13	.09	.13	−.13	.08	.097
Child gender (male)	.11	.08	.14	**.27**	**.07**	**<.001**
Length of stay in Canada	.07	.08	.40	−.06	.08	.43
Familial pre-migratory adversity	.19	.10	.06	**.24**	**.09**	**.005**
Emotion regulation	**−.51**	**.06**	**<.001**	**−.58**	**.07**	**<.001**
Familial Pre-Migratory Adversity x Emotion Regulation	−.13	.10	.17	**−.14**	**.07**	**.047**

Table S2. Sympathy regression models predicting child mental health for each adversity type

	**Internalizing**			**Externalizing**		
	β	*SE*	*p*	β	*SE*	*p*
**Child pre-migratory adversity model**						
Child age	.18	.10	.06	−.02	.09	.79
Child gender (male)	.11	.10	.25	**.18**	**.09**	**.045**
Length of stay in Canada	.05	.10	.62	.03	.10	.77
Child pre-migratory adversity	.04	.11	.74	.12	.09	.19
Sympathy	.08	.10	.40	−.13	.11	.23
Child Pre-Migratory Adversity x Sympathy	.05	.12	.68	−.14	.11	.17
**Parental pre-migratory adversity model**						
Child age	.17	.10	.08	−.04	.09	.68
Child gender (male)	.15	.09	.12	**.23**	**.09**	**.02**
Length of stay in Canada	.09	.10	.35	.03	.10	.76
Parental pre-migratory adversity	.18	.13	.15	**.19**	**.09**	**.04**
Sympathy	.08	.09	.38	**−.20**	**.10**	**.04**
Parental Pre-Migratory Adversity x Sympathy	−.04	.13	.77	−.08	.10	.40
**Familial pre-migratory adversity model**						
Child age	.18	.10	.06	−.04	.09	.69
Child gender (male)	.14	.09	.13	**.21**	**.09**	**.02**
Length of stay in Canada	.09	.09	.32	.05	.10	.64
Familial pre-migratory adversity	.23	.12	.06	**.21**	**.09**	**.02**
Sympathy	.10	.09	.24	−.16	.09	.08
Familial Pre-Migratory Adversity x Sympathy	.09	.14	.53	−.06	.10	.50

Table S3. Optimism regression models predicting child mental health for each adversity type

	**Internalizing**			**Externalizing**		
	β	*SE*	*p*	β	*SE*	*p*
**Child pre-migratory adversity model**						
Child age	**.18**	**.09**	**.05**	−.07	.09	.45
Child gender (male)	.03	.09	.74	**.18**	**.09**	**.05**
Length of stay in Canada	.06	.09	.52	−.04	.10	.67
Child pre-migratory adversity	−.01	.11	.90	.13	.10	.19
Optimism	**−.28**	**.09**	**.003**	**−.23**	**.09**	**.009**
Child Pre-Migratory Adversity x Optimism	.06	.12	.63	−.01	.10	.94
**Parental pre-migratory adversity model**						
Child age	**.19**	**.09**	**.03**	−.07	.09	.42
Child gender (male)	.09	.09	.33	**.24**	**.09**	**.007**
Length of stay in Canada	.09	.08	.25	−.03	.09	.73
Parental pre-migratory adversity	**.23**	**.11**	**.04**	**.20**	**.09**	**.02**
Optimism	**−.33**	**.10**	**.001**	**−.25**	**.10**	**.01**
Parental Pre-Migratory Adversity x Optimism	−.19	.11	.09	−.01	.10	.91
**Familial pre-migratory adversity model**						
Child age	**.20**	**.09**	**.02**	−.06	.09	.48
Child gender (male)	.08	.09	.40	**.22**	**.09**	**.01**
Length of stay in Canada	.16	.09	.09	.01	.10	.89
Familial pre-migratory adversity	**.36**	**.12**	**.003**	**.29**	**.12**	**.01**
Optimism	**−.31**	**.09**	**<.001**	**−.25**	**.09**	**.004**
Familial Pre-Migratory Adversity x Optimism	**−.31**	**.13**	**.02**	−.10	.12	.41

Table S4. Trust regression models predicting child mental health for each adversity type

	Internalizing			Externalizing		
	β	*SE*	*p*	β	*SE*	*p*
**Child pre-migratory adversity model**						
Child age	**.18**	**.09**	**.04**	−.07	.09	.42
Child gender (male)	.12	.09	.18	**.25**	**.09**	**.005**
Length of stay in Canada	.05	.09	.55	−.03	.09	.73
Child pre-migratory adversity	.09	.11	.40	.12	.11	.24
Trust	**.29**	**.09**	**.002**	.08	.09	.39
Child Pre-Migratory Adversity x Trust	−.15	.11	.17	.08	.10	.44
**Parental pre-migratory adversity model**						
Child age	.17	.09	.07	−.06	.09	.51
Child gender (male)	.18	.09	.06	**.29**	**.09**	**.001**
Length of stay in Canada	.09	.09	.31	−.03	.10	.78
Parental pre-migratory adversity	.23	.14	.11	.19	.11	.07
Trust	**.19**	**.09**	**.03**	.03	.10	.77
Parental Pre-Migratory Adversity x Trust	−.09	.13	.47	−.01	.11	.95
**Familial pre-migratory adversity model**						
Child age	**.17**	**.09**	**.06**	−.06	.09	.50
Child gender (male)	.14	.09	.11	**.27**	**.09**	**.002**
Length of stay in Canada	.10	.08	.25	−.003	.10	.97
Familial pre-migratory adversity	.25	.14	.08	**.22**	**.10**	**.03**
Trust	**.24**	**.09**	**.007**	.04	.09	.62
Familial Pre-Migratory Adversity x Trust	−.18	.14	.19	.02	.11	.84
